# Digital assessment of properties of the three different generations of dental elastomeric impression materials

**DOI:** 10.1186/s12903-022-02419-4

**Published:** 2022-09-05

**Authors:** Lamia Singer, Shaymaa I. Habib, Heba El-Amin Shalaby, Sayed H. Saniour, Christoph Bourauel

**Affiliations:** 1 Oral Technology, Medical Faculty, Dental School, University Hospital of Bonn, North Rhine-Westphalia, 53111 Bonn, Germany; 2 Department of Orthodontics, Medical Faculty, Dental School, University Hospital of Bonn, North Rhine-Westphalia, 53111 Bonn, Germany; 3 Dental Biomaterials Department, Faculty of Oral and Dental Medicine, Cairo University, Cairo, Egypt; 4 Dental Biomaterials Department, Faculty of Dentistry, October University for Modern Sciences and Arts, Cairo, Egypt; 5 Dental Biomaterials Department, Faculty of Oral and Dental Medicine, British University in Egypt, Cairo, Egypt

**Keywords:** Polyvinylsiloxane, Polyether hybrid, Hydrophilicity, Silicone elastomers, Dimensional accuracy

## Abstract

**Background:**

This study aimed to compare the dimensional accuracy, hydrophilicity and detail reproduction of the hybrid vinylsiloxnether with polyether and polyvinylsiloxane parent elastomers using modified digital techniques and software. This was done in an attempt to aid in solving the conflict between the different studies published by competitive manufacturers using different common manual approaches.

**Methods:**

A polyether, polyvinylsiloxanes and vinyl polyether silicone hybrid elastomeric impression materials were used in the study. Dimensional accuracy was evaluated through taking impressions of a metallic mold with four posts representing a partially edentulous maxillary arch, that were then poured with stone. Accuracy was calculated from the mean of measurements taken between fixed points on the casts using digital single-lens reflex camera to produce high-resolution digital pictures for all the casts with magnification up to 35×. Hydrophilicity was assessed by contact angle measurements using AutoCAD software. The detail reproduction was measured under dry conditions according to ANSI/ADA Standard No. 19 and under wet conditions as per ISO 4823. A metallic mold was used with three V shaped grooves of 20, 50, and 75 µm width. Specimens were prepared and examination was made immediately after setting using digital images at a magnification of 16×.

**Results:**

The hybrid impression (0.035 mm) material showed significantly higher dimensional accuracy compared to the polyether (0.051 mm) but was not as accurate as the polyvinyl siloxane impression material (0.024 mm). The contact angles of the hybrid material before and after setting was significantly lower than the parent materials. With regard to the detail reproduction, the three tested materials were able precisely to reproduce the three grooves of the mold under dry conditions. Whereas, under wet conditions, the hybrid material showed higher prevalence of well-defined reproduction of details same as polyether but higher than polyvinylsiloxane that showed prevalence of details with loss of sharpness and continuity.

**Conclusions:**

The digital technique used could be a more reliable and an easier method for assessment of impression materials properties. The hybridization of polyvinyl siloxane and polyether yielded a promising material that combines the good merits of both materials and overcomes some of their drawbacks.

## Introduction

The technical complexity in prosthodontic treatment has led to the development of indirect approaches in which the restoration is fabricated outside the oral cavity to be placed afterwards intraorally [1]. Accuracy of the final restorations depends on several parameters such as proper tooth preparation, operator and laboratory technician’s skills, and most prominently, the impression material and the technique used [2]. An ideal impression material should have an adequate flow, sufficient working and setting time, good dimensional accuracy and stability along with elasticity [3]. Over years, a wide variety of impression materials were developed, all trying to achieve an impression that can perfectly reproduce the oral hard and soft tissues. Although a number of materials and techniques have provided adequate clinical results, the ideal impression material has not yet been found [4, 5].Elastomeric impression materials are currently one of the most popular options for definitive impressions in fixed and removable prosthodontics [6]. A wide range of elastic impression materials are available for dental use that can be categorized into two groups, hydrocolloids and elastomers. Hydrocolloids are crucial material in every dental clinic with alginate being the most frequently used one. Alginate impression is usually used at the first dental visit to give an overview about the patient’s oral health status. The advantages of using alginate include the low cost, comfort of the patient, the speedy and easy manipulation, and possibility of obtaining a satisfactory primary impression in one step [7].

Elastomers or rubber base impression materials are the most widely used materials as secondary impressions as they meet most of the ideal requirements of an impression material. Among the available and the most frequently, used elastomers in the market are the polyvinyl siloxanes and polyethers [8]. Shrinkage on polymerization have long remained a major problem that affects directly the dimensional accuracy and stability of elastomeric impression material. Moreover, the distortion of the impression material during cast duplication of the same impression influences the accuracy and proper fit of the final restoration [9, 10]. The American Dental Association Specification for elastomeric dental impression materials endorses a maximum negative change in dimensions to be 0.50% after a minimum of 24 h [11].

Precise fit and adaptation of final restorations and appliances rely greatly on the ability of the impression material to reproduce all details of the soft and hard tissues. Clinically, the accuracy of the final restorations depends on two major aspects: the ability of the impression mixture to flow and adapt well to the relevant surfaces while taking the impression, and the wetting of the set impression by the gypsum on cast preparation [12]. However, under moist conditions, the accessibility of an ideal detail reproduction is even more challenging as several factors are involved [13], including wettability and rheological properties [2].

Polyvinylsiloxane (PVS) are dimensionally accurate and stable after setting, with an excellent ability to recover greatly from the deformation but they are hydrophobic [14]. This hydrophobic nature dictates the presence of a dry environment for achieving an accurate impression. Hydrophobicity is due to its chemical structure, which contains hydrophobic, aliphatic hydrocarbon groups surrounding the siloxane bond [15]. Consequently, certain non-ionic surfactants have been added to PVS materials and are described by the manufacturers as hydrophilic PVS materials. However, these modified PVSs are only slightly less hydrophobic than their precursors [14].

Polyethers (PE) are hydrophilic materials with dimensional stability and excellent detailed reproduction as main features. On the other hand, PEs have limitations of being expensive, highly stiff, difficult to be removed from the mouth, and possess an increased risk of die fracture when compared to polyvinylsiloxane [16]. Attempts to overcome the disadvantages have led to the introduction of the soft PE modified by decreasing the filler content to produce a less rigid material. The difficulties of removing the impression from a patient’s mouth and separating impressions from casts was reduced, especially in cases with significant undercuts. Another approach to reduce the stiffness of the polymerized material is by adding low viscosity softeners [17].

A novel guest elastomeric impression material, that combines the advantages of polyether and PVS materials and eliminates their drawbacks, has been introduced. It is being referred to as vinyl polyether silicone (VPES) hybrids [18]. The hybridization of PVS and PE and are available and marketed as hydrophilic materials (VPES) that presumably preserve the merits and features of the parent products. A hydrophilic material is provided with the polyether group without the use of a surfactant. Moreover, a siloxane groups that provides a dimensionally stable material is combined with polyether properties. Therefore, this material has been claimed to have the tear strength and dimensional stability of PVS, and yet the wettability and flow of polyether. The hybrid material has a platinum catalyst, and the setting reaction is contaminated when powdered gloves are used to mix the material [19, 20].

There is still a disagreement in literature regarding the superiority of dimensional accuracy and hydrophilicity among the various old and modified elastomeric impression materials. Moreover, analog methods documented in literature for the assessment of impression material properties has not been updated since years. Hence, the aim of this study was to compare the dimensional accuracy, hydrophilicity and detail reproduction under dry and moist conditions of polyvinylsiloxane, polyether and VPES hybrid materials using digital techniques.

## Materials and methods

### Materials

Three elastomeric impression material with their corresponding tray adhesives were used: polyether impression material (Impregum Penta S 3M ESPE, St. Paul, MN, USA), polyvinylsiloxane impression material (Express XT, 3M ESPE, Seefeld, Germany), vinylpolyether silicone (VPES) hybrid impression material (Identium impression material, Kettenbach GmbH & Co, Im Heerfeld, Germany). Mechanical mixing of polyether and vinylpolyether silicone was done using Pentamix 3 (3M ESPE, St. Paul, MN, USA) while, polyvinylsiloxane was mixed using garant dispenser.

### Methods

#### Dimensional accuracy

**Fig. 1:**
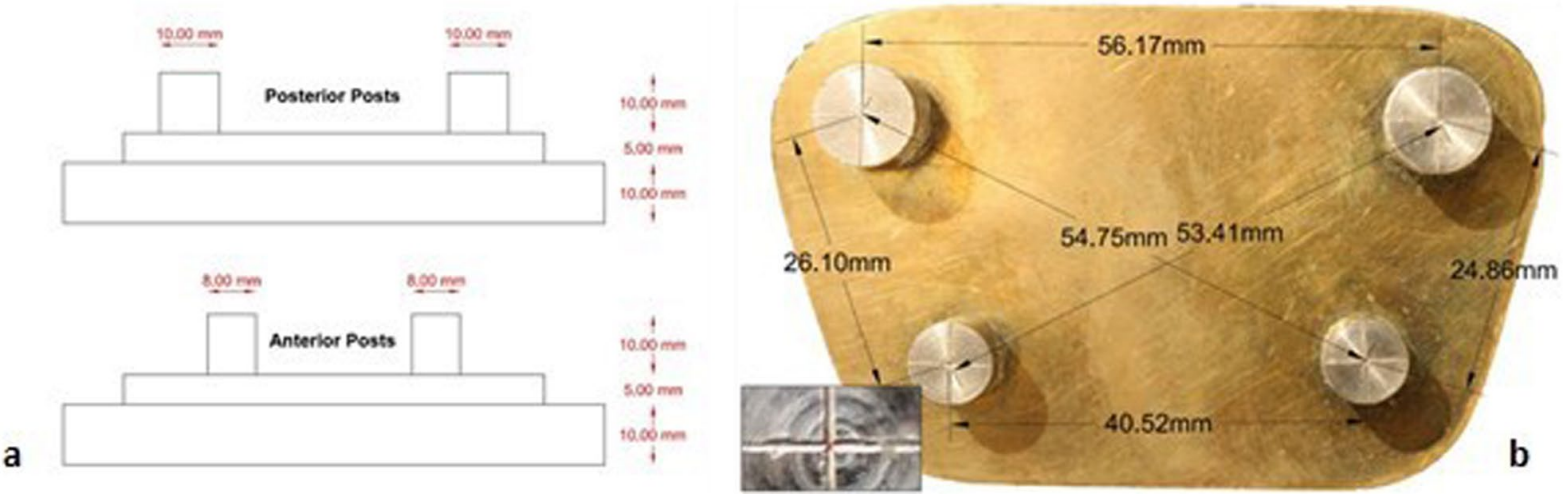
**a** a schematic diagram, **b** an illustration of the master model with dimensions

A metallic master model was designed to be a geometric representation of a partially edentulous maxillary arch (Fig. 1a, b). The base of the master model consisted of an upper small one over a lower large one to provide a definite stop on which the impression tray could be accurately seated. Both of the two bases were made of the same trapezoidal shape where the thickness of the small base was 5 mm and the large base was 10 mm. Four posts were then made, two anterior representing the premolars and two posteriors representing the molars. Geometric center of the upper surface of each post of the master model was cross-marked as a reference points to allow accurate measurement.

#### Tray and cast preparation

Five identical trapezoid shape metal trays with the same dimensions of the prepared aluminium model were machined to act as trays for the impression materials. The exact positions of the centers of the posts were located in the tray, and the four cylindrical holes were drilled around these centers with 16 mm diameter for the anterior holes and 18 mm diameter for the posterior holes. This allowed 4 mm clearance for the thickness of the materials around the posts.

Trays were loaded with the impression material and placed over the master model with pressure until attaining metal to metal contact, after setting of the impression, both tray and the model were attached to the upper and lower grips of a universal testing machine (Model LRX – plus: Lloyd, Farcham, UK) respectively. Universal testing machine was used for separating the tray from the model at a standardized crosshead speed of 0.5 mm\min and perpendicular to the model.

To form the working casts, Type IV gypsum (Zhermack; Whip Mix, Rovigo, Italy) was mixed with 20 mL of distilled water, first by hand for 15 s, and then vacuum-mixed for an additional 30 s (Degussa MultiVac Compact Vacuum Mixer, Germany). The gypsum was vibrated into the impression, filled to the level of the tray borders, and the excess material utilized to provide mechanical retention. The poured cast was left for 60 min at room temperature to ensure complete setting of the gypsum. To enable ease of separation of the gypsum working die, a base was added to the cast using a Type III dental stone (Zhermack; Whip Mix, Rovigo, Italy). The recovered gypsum cast with base was separated from the impression and left to set for 24 h in ambient air [21].

#### Dimensional accuracy measuring procedures

A Canon T3i (Canon Corp, NY, USA) digital single-lens reflex camera with 18–55 mm lens was used to produce high resolution digital pictures for all the casts, which allows magnification up to 35×. The camera was fixed on a tripod to standardize the shooting angle, and to standardize the distance between the camera lens and the casts. Additionally, a base was prepared with reference points to ensure standardization of the casts' placement. High resolution digital pictures were transferred to a computer, and AutoCAD 2012 (Autodesk Inc, CA, USA) software was used to measure the different dimensions. It is worth mentioning that when the engraved lines on the cast were magnified and viewed on the computer software, each line appeared as a groove having two edges. Therefore, a line was drawn between each two opposite edges and the point of intersection of these two lines was taken as a reference point in order to standardize the measuring procedure. The dimensional accuracy was evaluated by measuring each dimension on the master model and comparing it with the corresponding dimension on working casts [22].

#### Hydrophilicity

**Fig. 2:**
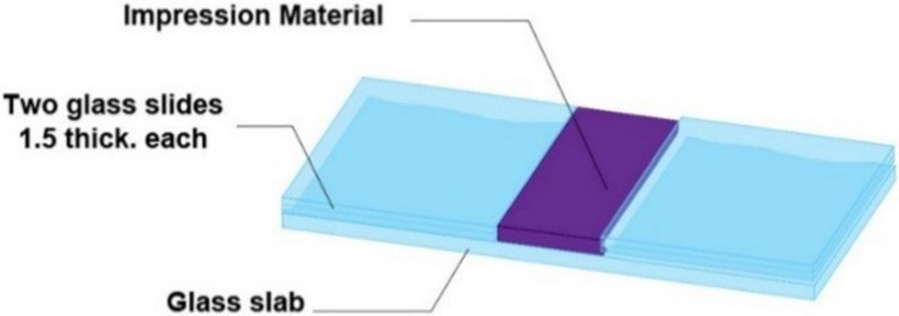
Schematic drawing for the contact angle mold used

Contact angle measurements were used to assess elastomers hydrophilicity. A glass plate served as a base and then two thin glass slides (3 mm thickness) were glued on either side of the glass base to act as a stopper [23]. All of the three elastomeric impression materials were mixed using the mixing machine recommended by their manufacturer with their corresponding supplied mixing tips. The first few centimeters of mixed paste were discarded to ensure complete homogenous mix. The impression material was spread out on the glass base and a uniform thickness of the material was obtained by the two side glass stoppers (Fig. 2).

**Fig. 3:**
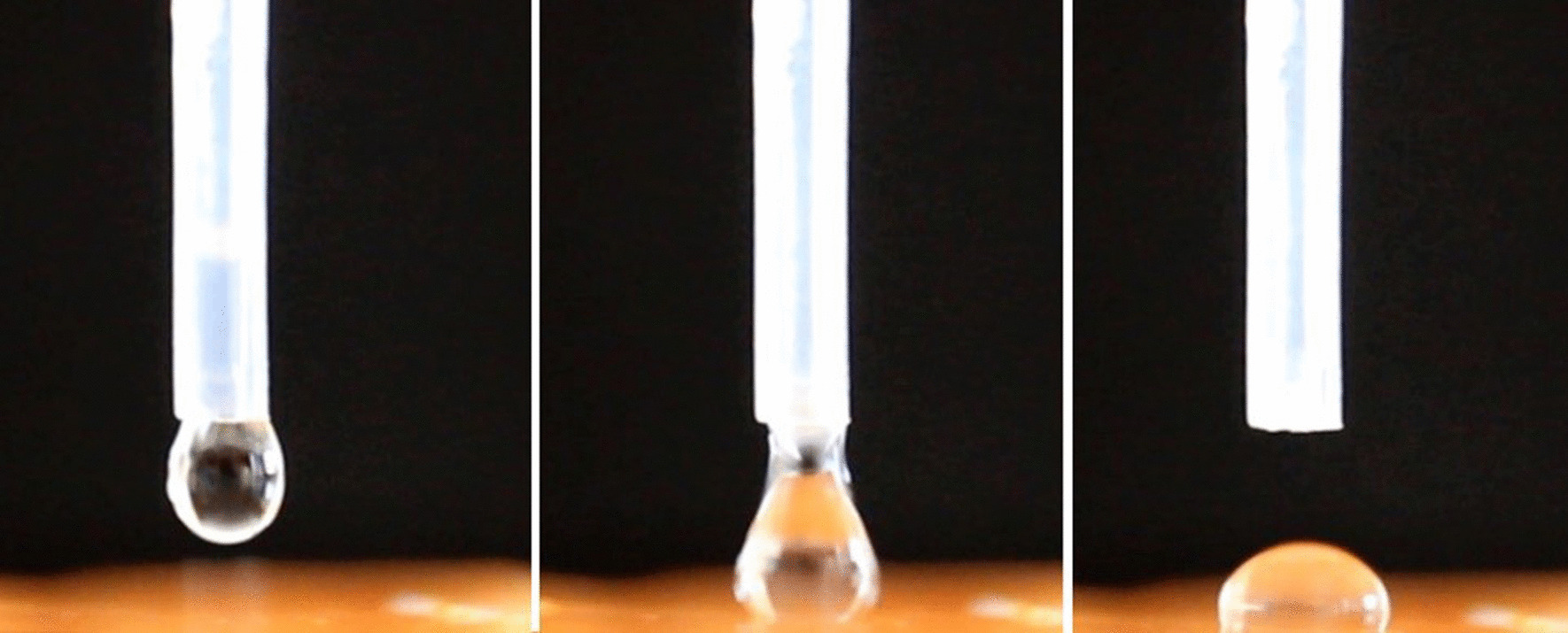
The water droplet application

The glass slab with the material was placed immediately 5 mm beneath a clamped water droplet calibrated pipette [23]. The time elapsed between application of the material to the glass slide and the start of the measurement was 50 s. The imaging by video capture was started before the droplet touched the surface to record the whole process of jump to contact (Fig. 3).The following developments of the contact angle were measured one minute from start of mixing (first drop) and the drop was monitored every 10 s till the end of the working time.The test was repeated at the end of the working time (second drop) indicated by the manufacturer for each material using Canon T3i (Canon Corp, USA) digital single lens reflex camera, with a Canon EF 100 mm macro lens. The camera was fixed on a tripod. A Lever rule was used to align both the camera and the specimens to ensure they were horizontal.

**Fig. 4:**
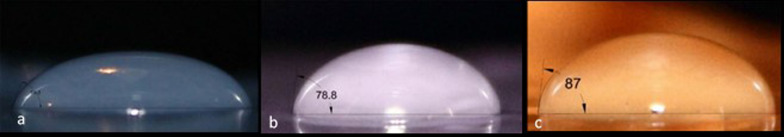
Measuring contact angle on **a** VPES, **b** PE, and **c** PVS specimens

The contact angle values (the angle between the tangent of the symmetrical water droplet and the interface between the droplet and the surface of the impression material) for all specimens were calculated with the use of Auto CAD 2012 (Autodesk Inc, CA, USA) software (Fig. 4a–c) [24]. All values gained in this experiment were relative measurements and can therefore only be used in this or similar experimental setups to compare the materials under investigation with each other.

#### Detail reproduction

The detail reproduction was assessed under dry conditions according to ANSI/ADA Standard No. 19 [25] and under wet conditions as per ISO 4823 [26]. Stainless-steel ruled block (A) with three V-shaped grooves, and a ring mold (B) with a metal plate (C) were used. The three V shaped grooves were 25 mm long and having varying widths of 20, 50, and 75 µm [25]. The impression materials were prepared according to their manufacturer’s instructions, placed inside the stainless-steel ring mold and slightly over filled. For moisture condition simulation, a fine mist of water was sprayed to the surface of the test block before applying the impression material onto it [14, 27]. After 1.5 min from start of mixing, the mold was immediately covered by the flat metal plate (C) and a 1 kg weight was applied over it for proper seating of the material. The material was placed in a water bath (32 ± 2 °C) and was then separated from the mold after three minutes from the minimum intraoral setting time recommended by the manufacturer.

Examination was made immediately using A Canon T3i (Canon Corp, NY, USA) digital single-lens reflex to produce high resolution digital pictures of specimens, at a magnification of 16×. The distance between the camera lens and the specimens was standardized using the same technique mentioned before. [28]. Ten specimens of each material were prepared and assessed as follow;

- Under dry condition (*n* = 5): the continuous replication of at least 2 of the 3 horizontal lines was evaluated [20].
- Under wet condition (*n* = 5): The reproduction of line (20 µm) was taken as a minimum requirement to pass or fail a specimen, then the entire length of the 20 µm line was observed and given an ordinal score as follows [29]:
  1. Well defined, sharp, continuous line.
  2. Continuous line but with some loss of sharpness.
  3. Significant deterioration of edge detail or loss of continuity of the line.
  4. Failure to reproduce the line.

## Statistical analysis

Quantitative variables showed parametric distribution and data were presented as mean and standard deviation (SD) values. One-way Analysis of Variance (ANOVA) was used for comparison between the three groups. Pair-wise comparison between the groups were done by Tukey’s post-hoc test when ANOVA test was significant. The significance level was set at *p*-value ≤ 0.05. Statistical analysis was performed with IBM (IBM Corporation, NY, USA) SPSS (SPSS, Inc., an IBM Company) Statistics Version 20 for Windows.

## Results

### Dimensional accuracy

**Fig. 5:**
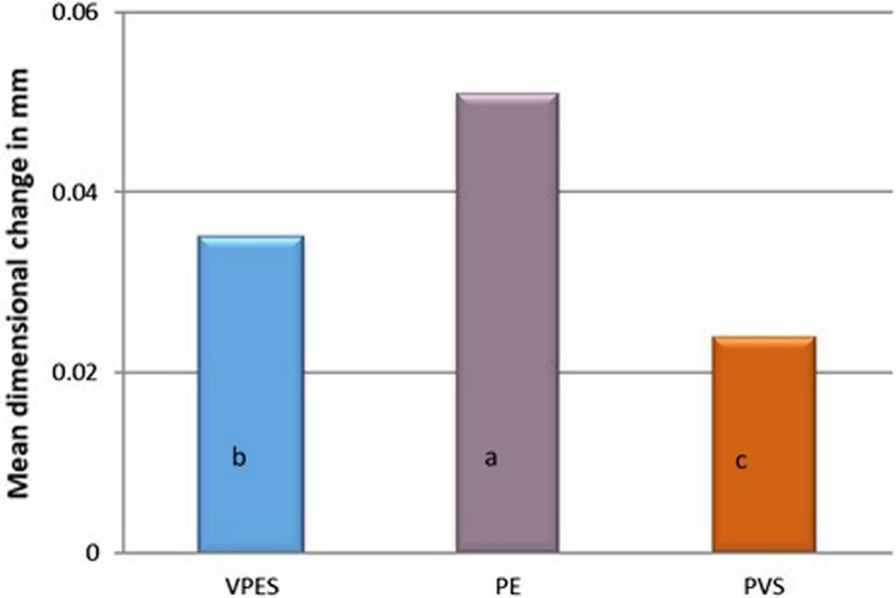
Bar chart representing the mean dimensional changes in mm for the three tested materials

Statistical analysis of the mean and standard deviation values of the dimensional changes of the tested materials are represented in Fig. 5. Based on the findings, an analysis of variance (ANOVA) indicated there was a clear statistically significant difference between dimensional changes in the three materials (*p*-value < 0.001). Post hoc comparisons using Tukey’s test indicated that polyether recorded significantly the highest mean dimensional changes (M = 0.051 mm, SD = 0.001). However, vinylpolyether silicone hybrid showed lower mean dimensional changes (M = 0.035 mm, SD = 0.0004) compared to PE but was higher than polyvinylsiloxane, that showed statistically the lowest mean values (M = 0.024 mm, SD = 0.001).

### Hydrophilicity

**Fig. 6:**
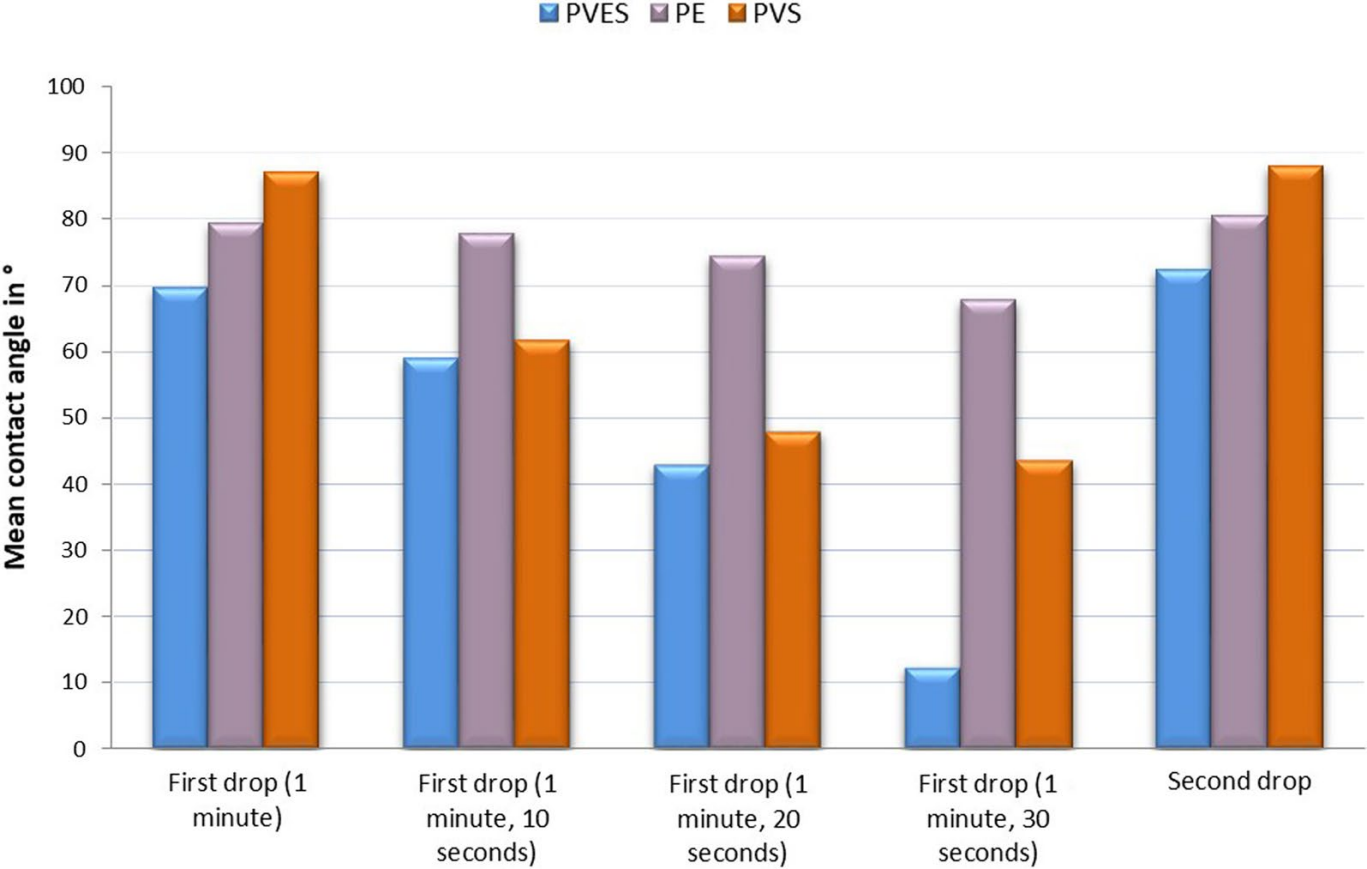
Bar chart representing the mean contact angles of the three tested impression materials

The variables showed parametric distribution and thus one way ANOVA was used followed by Tukey’s post hoc for pairwise comparison between the tested groups. Statistical analysis of the means and standard deviation of the hydrophilicity for the three tested materials at different times are represented in Table 1 and illustrated in Fig. 6.

**Table 1:** The mean, standard deviation (SD) values and results of One-way ANOVA test for comparison between contact angles in the three tested materials

	Vinylpolyether silicone hybrid (VPES)		Polyvinylsiloxane (PVS)		Polyether (PE)		*p*-value
	Mean (°)	SD	Mean (°)	SD	Mean (°)	SD	
First drop (1 min)	69.6^c^	1.1	87^a^	1.9	79.2^b^	1.1	< 0.001
First drop (1 min, 10 s)	59^c^	1.0	61.6^b^	2.4	77.8^a^	0.8	< 0.001
First drop (1 min, 20 s)	42.8^c^	0.8	47.8^b^	1.9	74.4^a^	1.7	< 0.001
First drop (1 min, 30 s)	12.2^c^	0.8	43.4^b^	1.14	67.8^a^	1.5	< 0.001
Second drop	72.2^b^	0.8	87.8^a^	1.48	80.4^b^	1.1	< 0.001

*p ≤ 0.05, Different letters are statistically significantly different

#### First drop

The contact angle was measured one minute from the start of mixing and the drops were monitored every 10 s until the end of the working time.

After 1 min; there was a statistically significant difference between the three materials (*p*-value < 0.001), where PVS showed significantly the highest mean contact angle (87), however polyether showed statistically lower mean contact angle (79.2), while VPES hybrid showed the lowest mean values (69.6).

After 1 min and 70, 80 and 90 s, there was a statistically significant difference between the three materials (*p*-value < 0.001). PE showed the highest mean contact angle (70 s = 77.8, 80 s = 74.4, and 90 s = 67.8). PVS showed statistically significantly lower mean contact angle (70 s = 61.6, 80 s = 47.8, and 90 s = 43.4). VPES hybrid showed the significantly lowest mean contact angle (70 s = 59, 80 s = 42.8 and 90 s = 12.2).

#### Second drop

A second drop measured the contact angle at the end of the working time indicated by the manufacturer for each material. There was a statistically significant difference between the three materials (*p*-value < 0.001), where PVS showed statistically the highest mean contact angle (87.8), however there was no statistically significant difference between VPES hybrid) (72.2) and PE (80.4).

### Detail reproduction

**Fig. 7:**
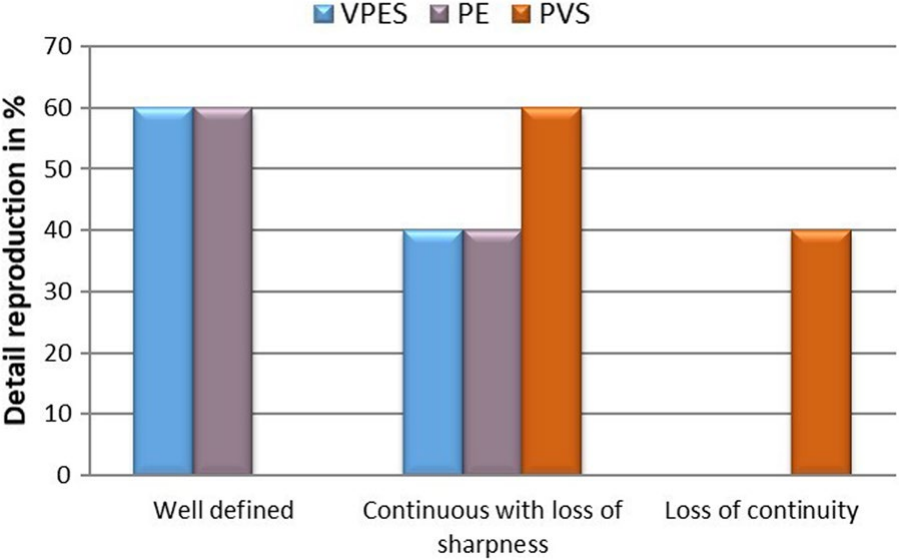
Bar chart representing detail reproduction in % for the three tested materials

The variables showed parametric distribution and thus one-way analysis of variance (ANOVA) was used to test the detail reproduction followed by Tukey’s post-hoc test for pairwise comparison between the tested groups for the wet condition. The means and standard deviation values of the detail reproduction for the three tested elastomeric impression materials under dry and wet conditions are represented in Fig. 7.

### Dry condition

The three materials were capable of recording the three lines over their full length; and therefore, there was no statistically significant difference between the three materials with regard to their ability to reproduce the details.

### Wet condition

Analysis of variance (ANOVA) revealed that there was a statistically significant difference between the three tested materials (*p*-value = 0.049), where VPES (60%) and PE (60%) showed higher prevalence of well-defined reproduction of details. However, PVS showed prevalence of details with loss of sharpness (60%) and continuity (40%).

## Discussion

### Dimensional accuracy

Dimensional accuracy of an impression material is a very important aspect when considering the replication process of the oral soft and hard tissues [27]. An impression accuracy depends on many factors, such as chemical composition, setting reactions, by-products, and disinfection [21, 30]. Although an exact dimensional replica may be very difficult as many of the steps result in dimensional changes, some of these changes might compensate at a certain point for one another [31, 32].

A metallic mold was used in the present study for evaluation of the accuracy to ensure dimensional stability of the master model during the whole testing period and under different environmental conditions. Moreover, type VI stone was used for preparation of the stone casts because it is highly compatible with elastomers and is considered very accurate in recording the fine details as well. In addition, to overcome the great deal of errors that usually result from the analogue method, a high-resolution digital camera with an AutoCAD software was used to measure the dimensional changes.

The results of this study showed that all the tested materials coincided with the ADA specification no. 19 that recommends a maximum negative change in dimensions to be 0.5% after a minimum of 24 h, and the ISO 4823 specification which allows a dimensional change of less than 1.5%. This was in agreement with previous reports stated by Mandioks M. et al. [33], Chai J. et al. [5], Faria A. et al. [9], Stober T. and Schmitter M [21] and Katyayan PA. et al. [34].

In the current study, the polyvinylsiloxane impression showed the highest dimensionally accuracy among all tested materials followed by VPES, whereas the PE showed the least accuracy. The high accuracy of PVS could be attributed to the excellent physical and mechanical properties of this type of material including high elastic recovery, and good tear strength [35]. Moreover, its setting occurs through an addition polymerization reaction between the silane and vinyl groups terminal groups with the hybrid group without the formation of by-product and material shrinkage [36].

Conversely, the newly formulated platinum-initiated vinylpolyether silicone (VPES) showed significantly lower dimensional changes compared to polyether that might be referred to its chemical composition. VPES consists of a copolymer of α-divinylpolydimethylsiloxane and α-divinyl polyether cross-linked by organohydrogenpolysiloxane [21]. This composition intended to incorporate the natural hydrophilicity of conventional polyether impression materials along with the desirable properties of vinyl polysiloxane materials [37]. For further wetting and flowability enhancement, the manufacturer has incorporated a surface tension eraser (STES) and wetting conditioner surfactant (WCS) into the vinyl siloxanether [36].

However, the high dimensional changes recorded by the PE group could be attributed to the ring opening cationic addition polymerization reaction. Each stage of the reaction and the ionized form of sulfonic ether acid involve the opening of an epimine ring. Each molecule of prepolymer has two reactive groups of epimine; therefore, induced propagation produces chain elongation, causing expansion [38].

### Hydrophilicity

According to O’Brien [39], wetting describes the relative affinity of a liquid for a solid, which can be quantified by measuring the contact angle. The result of the present study showed that the mean contact angle of VPES hybrids was (69.6); polyether was (79.2) and the mean contact angle produced by the hydrophilic polyvinylsiloxane (Express) was (87), one minute after mixing. This was in agreement with several studies [24, 40], which stated that polyether behaves more hydrophilic during the process of setting and can therefore exhibit better flow properties compared to PVS materials.

On monitoring the droplet every 10 s, polyvinylsiloxane had fast kinetics towards more hydrophilic equilibrium surface state compared to polyether. This possibly balances the disadvantage of the initial PVS impression materials hydrophobicity. Results of present study was similar to the conclusions drawn by Ruff et al. [41] and Kugel G et al. [42] who stated that, although surfactants were added to these materials, their hydrophilicity remained less than conventional polyether.

Polyether showed pronounced and constant initial hydrophilicity throughout the prescribed working time, which is consistent with Michalakis X et al. [13]**.** This could be due to the polar polyether molecule that provides the material with an inherent hydrophilicity and low contact angle. Polyether impression material is more hydrophilic because of its functional groups [carbonyl (C=O) and ether (C–O–C)]. These polarized groups can attract and interact with water molecules [43].

Conventional polyvinylsiloxane behaves hydrophobically because it does not contain any polarized groups. Nonionic surfactants have been incorporated into PVS to overcome the inherent hydrophobicity [43]. These surfactants act through a diffusion transfer of ionic molecules from the surface of PVS into the aqueous phase in order to reduce the surface tension of the liquid [44]. As for the surfactant once it reaches the surface, lee DY et al. [45] believe it remains attached to the impression surface, while others postulated that the surfactant is released into the liquid at the interface [46].

VPES hybrids wettability results from its chemical structure as they have polyether functional groups and probably also to surfactants added to the material. The chemical structure of the hybrid material as claimed by manufacturers is formed of large polyether molecules as a backbone to which smaller polyvinylsiloxane molecules are attached [43].

VPES results in the present study reinforce Menees et al. findings [47]. However, these results are not consistent with another study which claimed that only soft PE material is intrinsically hydrophilic (initial contact angle < 90°), whereas VPES is intrinsically hydrophobic (contact angle > 90°) [48].

Additionally, it was found that the time of the droplet placement in relation to the working time affects the wettability, where for additional silicone higher contact angles were observed at the end of the working time. This can also be explained on the same concept of surfactant migration as it may be difficult for the surfactant to migrate while polymerization and cross linking proceeded at the end of the working time [49].

On the other hand, polyether and VPES hybrid reported higher contact angle at the end of the working time compared to that obtained one minute after mixing. This could be attributed to the mobile polymer chains at the start of the working time, however at the end of the working time, the polymerization reaction and the polymer network formation would have progressed enough to cause more steric hindrance that decrease the interaction with water molecules [50–52].

### Detail reproduction

With regard to detail reproduction, all the three tested impression materials in this study reproduced the three lines under dry conditions. Therefore, the three impression materials complied with the ADA requirements for detail reproduction of elastomeric impression materials. These results are in agreement with Petrie CS. et al. [53], who concluded that the best surface details were obtained only under dry conditions for elastomeric impression material**.**

Under wet condition, there was a significant difference among the three tested impression materials regarding definition, sharpness and continuity of the 25 μm line. VPES hybrid and PE showed higher prevalence of well-defined reproduction of details. However, PVS showed higher prevalence of continuous details with loss of sharpness. Additionally, PVS was the only material that showed loss of continuity.

These results were in agreement with German et al. [2], who concluded that the hydrophilic polyether material is capable of reproducing fine details on moist surfaces while, many polyvinylsiloxane products are not capable of yielding the same results. This could be due to hydrophilicity, which is the main property that affects wetting of oral soft and hard tissues and correspondingly, affects the accurate detailed reproduction of prepared tooth surfaces [54, 55].

The inherent hydrophilicity of polyether accounted for its superior behaviour in moist conditions, however for vinylpolyether silicone hybrids the presence of both polyether and hydrophilic addition silicone in its chemical composition are responsible for the reasonable detail reproduction supported by the adequate hydrophilicity.

Results of this investigation reinforce postulations stating that the so-called hydrophilic polyvinylsiloxane material remains hydrophobic in the un-polymerized, liquid state and will not adequately wet moisturized surfaces [40, 46]. Though incorporation of hydrophilic surfactants, which commonly consist of an oligoether or polyether substructure, improves the distinct hydrophobicity of conventional PVS, it appears that the impression material still cannot accurately reproduce the details in the presence of moisture [56, 57].

## Conclusions

Within the context of this study, it can be concluded that dimensional accuracy of polyether was improved by its hybridization with addition silicon, which was expressed in lower values of vinylpolyether silicone hybrids dimensional changes compared to polyether. The hybrid material showed better hydrophilicity and lower contact angles before and after setting. Vinyl polyethersilicone hybrid elastomeric impression material showed detail reproduction comparable to polyether and better than addition silicon elastomeric impression materials under wet conditions. Moreover, the dryness and wetness of the surface has a great impact on the detail reproduction of the different investigated materials. Future work involving comparing digital cameras, scanners and micro computed tomography analysis are planned for a better over view.

## Data Availability

The datasets used and/or analysed during the current study are not publicly available due to minor restrictions but are available from the corresponding author on reasonable request.
